# Engineering Extracellular Microenvironments: The Impact of Fibrous Materials on Cell Behavior

**DOI:** 10.1002/adhm.202501942

**Published:** 2025-08-11

**Authors:** Zan Lamberger, Gregor Lang

**Affiliations:** ^1^ Department of Functional Materials in Medicine and Dentistry University of Würzburg Pleicherwall 2 97070 Würzburg Germany

**Keywords:** 3D models, artificial matrices, electrospinning, fibers, melt electrowriting

## Abstract

The development of tissue models and replacements that closely mimic native biological structures is a central goal in tissue engineering and biofabrication. These models aim to reduce animal testing and improve the relevance and translatability of experimental results. A key step is the transition from simple two‐dimensional cultures to three‐dimensional systems that better reflect the architecture of the extracellular matrix. Replicating the hierarchical organization of native tissues is essential, particularly the fibrous networks mainly composed of collagen, which regulate cell alignment, migration, proliferation, and differentiation. Incorporating such structures has proven highly effective and often necessary to induce cell behaviors resembling those in vivo. This review first examines the cellular mechanisms that govern interactions with fibrous microenvironments. It then outlines key design parameters for fiber‐based substrates, including chemical composition, diameter, surface topography, and alignment. These factors can be tuned to guide cell organization and function. Strategies for translating these principles into three‐dimensional fiber‐reinforced constructs and bioinks are then discussed, with a focus on current approaches for creating biomimetic environments. The article concludes with future perspectives, highlighting the potential of fibrous scaffolds and advanced fabrication techniques to enable next‐generation tissue models and regenerative therapies.

## Graphical abstract

Fibrous structures are key elements of the native extracellular matrix and crucial for directing cell behavior. This review discusses how fiber properties such as composition, diameter, and alignment affect cell responses in 2D and 3D systems. Strategies for integrating fibrous cues into engineered tissues are highlighted, and future directions for scaffold design in regenerative medicine are explored.

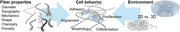

## 1 Introduction

Cells are simultaneously the engines, engineers, and building blocks of all simple and complex biological lifeforms. They continuously reassemble matter into new functional units at multiple scales, from molecules up to tissues, organs, and entire organisms.^[^ ^1^, ^2^, ^3^ ^]^ Ever since discovering how these tiny building blocks create such complex structures, humans have been intrigued, and often puzzled, by the underlying mechanisms in a quest to understand, control, and harness their potential to drive them beyond the constraints of regular evolution.^[^ ^4^, ^5^, ^6^ ^]^ A crucial step is to decipher the complex interplay of factors and interactions that guide cell differentiation, proliferation, and tissue maturation. This has been an effort of decades, if not centuries, ever since controllable tissue regeneration/reconstruction and targeted disease therapy became a tenable idea, and many steps and leaps have been made in this direction.^[^ ^7^, ^8^ ^]^

The instructions for cell function lie within their DNA and partly in inherited organelles.^[^ ^9^, ^10^, ^11^ ^]^ In the zygote, the components have the potential to form any cell in the body. Through replication and environmental cues, gene expression becomes increasingly specialized, resulting in diverse cell types. This process is known as differentiation.^[^ ^12^, ^13^ ^]^ Cellular differentiation and behavior are influenced by various external factors, including soluble molecules such as growth factors and hormones, as well as other receptor‐interacting molecules.^[^ ^14^ ^]^ On the other hand, the overall surface chemistry and structure on which they reside, adhere to, and migrate on also play important roles.^[^ ^15^, ^16^ ^]^ All these processes are regulated by interactions with bioactive molecules produced, released, or presented by cells to communicate, sense their surroundings, and coordinate their growth, migration, proliferation, and differentiation.^[^ ^17^ ^]^ To drive further functional hierarchy, such as that needed for tissue and organ development in terms of cellular adhesion, tissue mechanics, and other means of cell signaling, some cells begin to produce and remodel the extracellular matrix (ECM).^[^ ^18^, ^19^ ^]^ This protein/polysaccharide‐based network with a high water content, i.e., a hydrogel, further guides and modulates cell behavior. It provides mechanical stability and ensures the spatial separation of tissues and organs, driving tissue organization during development and preserving structural heterogeneity and anisotropy in mature tissues.^[^ ^20^, ^21^ ^]^ Understanding its function and mimicking it is vital for the purposes of many medical fields, whether for developing disease models, drug/cancer screening devices, or tissue replacement in regenerative medicine.^[^ ^22^, ^23^ ^]^ The most important ECM hydrogel matrix components are proteins, of which ≈300 different types have been identified, many forming glycoproteins or proteoglycans through conjugation with saccharides. Combined interweave to create the ECM's hierarchical fibrous architecture, mainly driven by collagen. This fibrous network is essential for regulating mechanical properties, cell guidance, and partly for cell‐cell signalling and interactions.^[^ ^20^, ^24^ ^]^

In tissue engineering and biofabrication, polymer‐based fibers are increasingly used to mimic the complex fibrous structures of the native ECM. By replicating the hierarchical organization of fibrils and fibers, it becomes possible to recreate the physical, chemical, and mechanical cues that cells rely on to sense their environment and respond appropriately.^[^ ^25^, ^26^ ^]^ In this review, fibrils are defined as nanoscale, thread‐like structures with diameters of ≈1 to 100 nm that can assemble into larger fibrous networks,^[^ ^27^ ^]^ while fibers are larger, elongated structures with a high aspect ratio that may consist of bundled fibrils or exist independently. Such polymeric fiber‐based scaffolds should not only provide structural support but also actively modulate cellular functions, guiding processes like tissue formation, regeneration, and remodelling.^[^ ^28^ ^]^ Understanding how to design and use these synthetic fibrous materials is therefore essential for developing advanced, biomimetic extracellular microenvironments that activate and control specific biological responses. In the following sections, we will explore the fundamental mechanisms by which cells interact with fibrous structures and discuss how engineered fibrous materials can be designed to replicate the complex functions of the native ECM.

## 2 Fundamentals of Cellular Interactions

To understand tissue development and cellular behavior, it is crucial to comprehend cellular interactions with their microenvironment. Cells possess an arsenal of transmembrane proteins to interact with their environment.^[^ ^29^ ^]^ Based on the constituents present on the membrane and within the cytoplasm, they process environmental cues and initiate or regulate signaling cascades. These alter the cell's biochemical makeup and influence cellular behavior, generating an integrated response.^[^ ^30^ ^]^

In addition to cell–cell communication via soluble signaling molecules, dynamic cell–cell and cell–matrix interactions play a central role.^[^ ^31^ ^]^ Adhesion is essential for cells to integrate into tissues and fulfill their functions. This involves three main types of interactions that need to be understood: adhesion between cells, adhesion of cells to surfaces, and cell migration. In the context of cell adhesion, this process is associated with the dynamic assembly and disassembly of protein complexes that span the cellular membrane, forming mechanical linkages between the cell's extracellular environment and the cytoskeleton. These linkages regulate the firmness of adhesions, the types of cellular connections, and migration rates.^[^ ^32^ ^]^

### 2.1 Cell–Cell Adhesion

Besides cell–surface/ECM connections, an equally important aspect is cell–cell contact, which is essential for tissue integrity, especially in tissues with high cell density. The most common types of cell–cell contacts are adherens junctions, desmosomal junctions, occluding junctions, and gap junctions. These serve vastly different purposes and are composed of distinct molecular constituents, which are described in the following.

#### 2.1.1 Adherens Junctions

**Figure 1:**
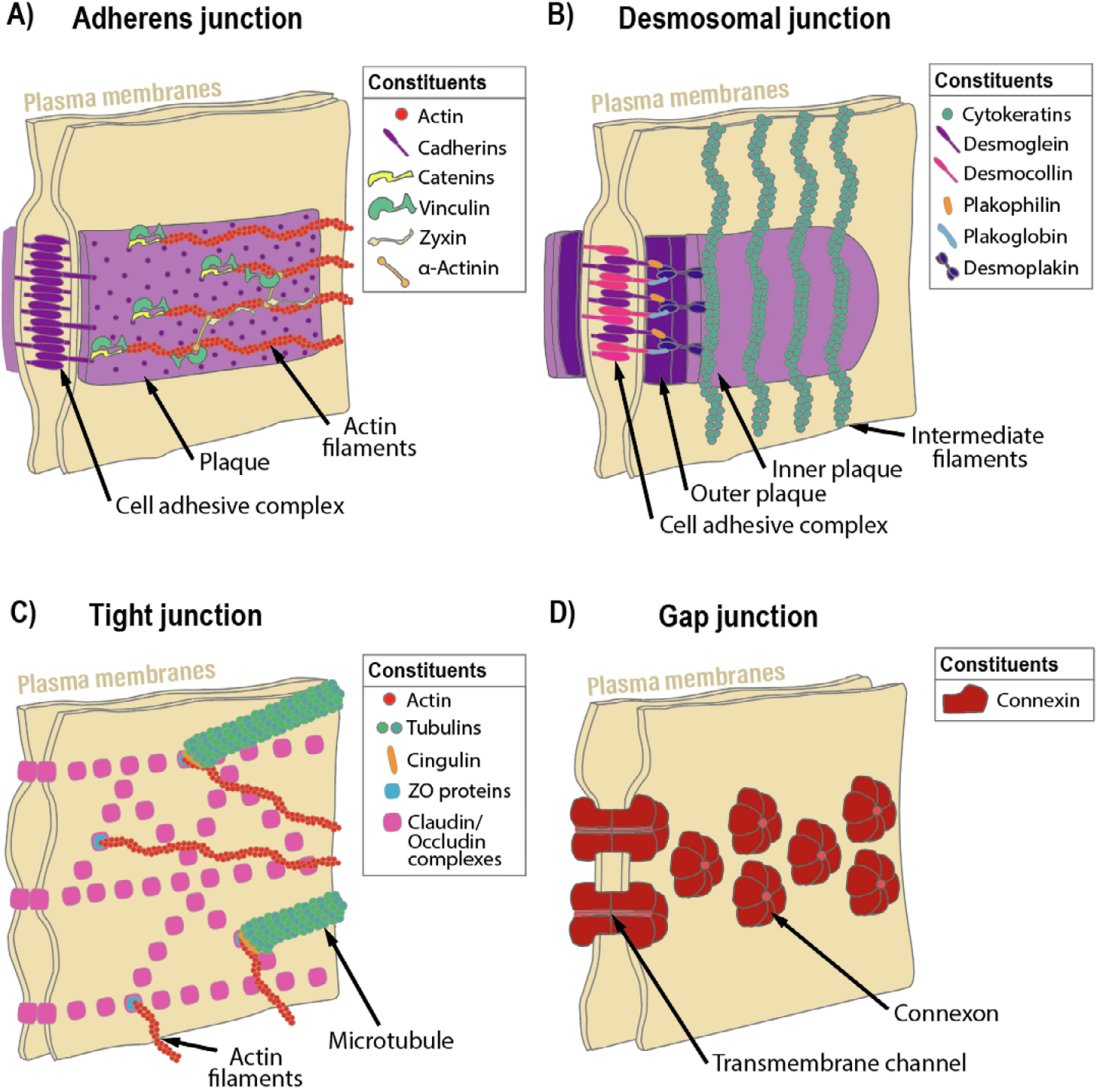
Schematic representations of cell‐cell adhesions, showing their most essential constituents and their likely arrangements, as shown for A) adherens junctions, B) desmosomal junctions, C) tight junctions, and D) gap junctions.

Among the most common cell–cell adhesive junctions are adherens junctions (**Figure** **1**A). These are formed by Ca^2^⁺‐dependent transmembrane cadherin proteins, which form complexes with cadherins presented by neighboring cells, resulting in a zipper‐like adhesion complex.^[^ ^33^ ^]^ Within the cytoplasm, actin filaments begin to assemble around the anchored cadherin complexes via associated plaque proteins. These filaments connect the junctions to the broader actin–tubulin cytoskeleton, providing additional stabilization when required.^[^ ^33^ ^]^ Adherens junctions are common in epithelia and form the foundation for the development of other types of cell junctions. They are essential for maintaining tight and interconnected tissue layers. Conversely, the loss of cadherin expression is associated with increased cell invasiveness and metastasis, as cadherins normally act to suppress cell motility.^[^ ^34^ ^]^

#### 2.1.2 Desmosomal Junctions

Desmosomes are specialized cell–cell adhesive structures that provide mechanical stability to tissues exposed to significant physical stress. They are composed of desmogleins and desmocollins (Figure 1B), which are transmembrane adhesion receptors and members of the cadherin superfamily. On the cytoplasmic side, desmogleins and desmocollins are anchored to a dense network of cytoplasmic plaque proteins, including plakoglobin, plakophilins, and desmoplakin. These plaque proteins link the desmosomal cadherins to the intermediate filament cytoskeleton, typically composed of keratin in epithelial cells or desmin in cardiac muscle cells.^[^ ^35^, ^36^ ^]^ Together, these components form a robust intercellular network that distributes mechanical stress across cells and enhances tensile strength throughout the tissue. Desmosomes are especially abundant in epithelial tissues (e.g., skin) and cardiac muscle, where they are crucial for maintaining structural integrity during stretching and contraction.^[^ ^37^ ^]^

#### 2.1.3 Occluding Junctions

Also known as tight junctions, these junctions enable the creation of pores in the spaces between cell layers for selective permeability of molecules and even other cells. They are composed of several transmembrane proteins, and their composition depends on what these junctions should be permeable to. The main components of these junctions are claudins and occludins, which associate with other plaque proteins and additional protein building blocks that vary depending on the junctions’ function (Figure 1C). The plaques are connected to the actin filaments and microtubules of the cell cytoskeleton.^[^ ^38^ ^]^ Although there are models, the exact compositions of tight junctions are not yet fully understood, but are not relevant for the scope of this review. In this case, the initial assembly requires E‐cadherin.^[^ ^39^ ^]^

This cell–cell adhesion complex mainly fulfills the dual purpose of regulating permeability between adjacent cells based on size and charge. It can also function to divide the cell membrane into two distinct surface regions, as membrane‐bound components cannot diffuse past the tight junction.^[^ ^40^ ^]^ These junctions are found in all tissues that create selectively permeable barriers, such as endothelia, epithelia, the intestine, and the blood–brain barrier. The permeability characteristics of the junctions naturally vary from tissue to tissue.

#### 2.1.4 Gap Junctions

These are clusters of channels connecting adjacent cells that enable the direct diffusion of ions and small molecules. They are formed by the head‐to‐head association of hexameric transmembrane complexes termed connexons, each composed of connexins (Figure 1D), localized on the membranes of the associating cells.^[^ ^41^, ^42^ ^]^ These channels then cluster into larger plaques containing approximately a thousand channel units. Due to the very close membrane association required for these junctions, only a very thin ≈2 nm gap remains between the cells at those points. These junctions are essential for the rapid exchange of ions, which is crucial for electrically excitable cells such as neurons and myocytes. However, they are also present in most cells that comprise solid tissues, where their primary role is to coordinate metabolic activity among groups of cells and thus help buffer gradients of nutrients and signaling molecules.^[^ ^43^ ^]^

### 2.2 Cell–Surface Adhesion and Migration

**Figure 2:**
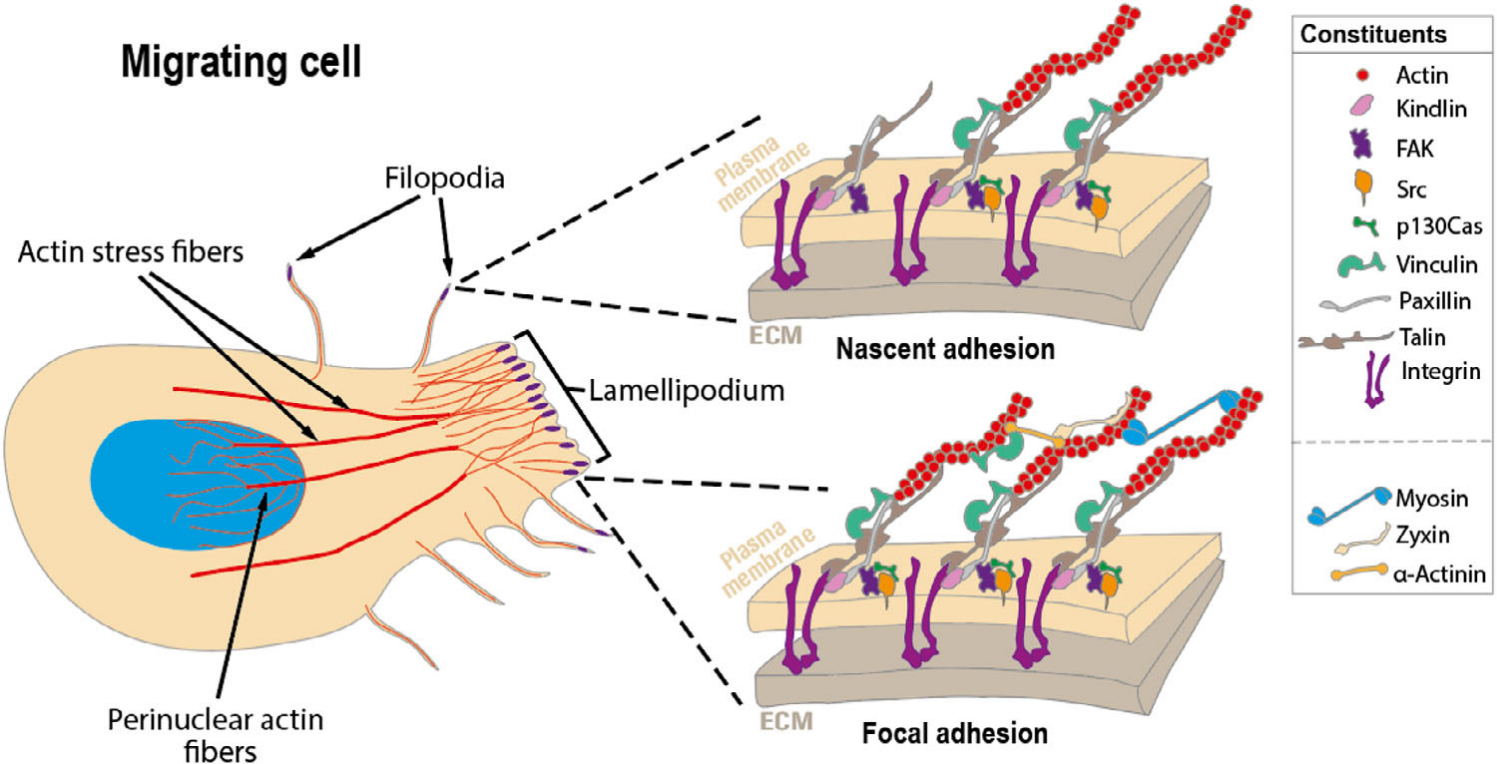
Representation of a migrating cell showing the most typical structures used for cell migration when interacting with the ECM, with enlarged schemes showing the main cellular protein constituents of nascent and focal adhesions.

Cell migration and adhesion vary widely, from slow movement with strong attachments to fast movement with weaker ones, depending on the cell type and mechanism. Cells respond to different signals: chemical, mechanical, or topographic. Some signals are soluble, like cytokines or growth factors that guide cell movement (chemotaxis), while others come from physical stimuli like stretching, flow, or electrical signals.^[^ ^44^ ^]^ When the cues are presented on a surface, cells usually sense these by extending parts of their actin cytoskeleton. They first use filopodia, thin membrane extensions supported by actin, to sense the surface. If the filopodia attach successfully, the cell's membrane starts to polarize and forms larger, sheet‐like extensions called lamellipodia (**Figure** **2**). These are stabilized by new adhesions, which begin as small, short‐lived integrin‐based contacts (nascent adhesions or focal complexes) and can develop into stronger focal adhesions in favorable areas. Conjoined with contraction of the cell's actomyosin machinery and connections to other actin structures like stress fibers,^[^ ^45^ ^]^ the cell body moves forward. Simultaneously, actin and adhesion depolymerization occur in less favorable areas, guiding the cell along favorable chemical or surface cues.^[^ ^46^ ^]^

#### 2.2.1 ECM Adhesion

**Figure 3:**
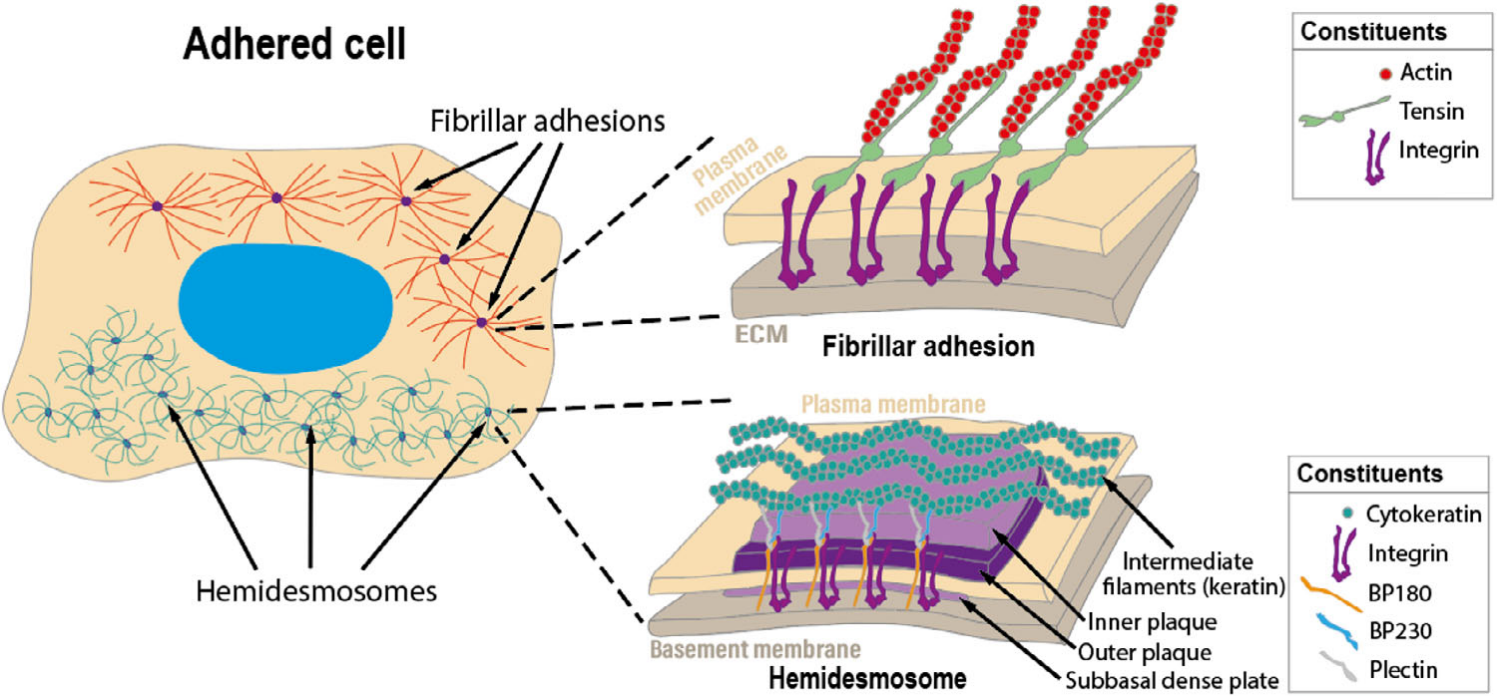
Representation of an adhered cell, showing structures typical for strong cell‐surface adhesion, with the main cellular protein constituents of fibrillar adhesions and hemidesmosomes illustrated.

When these cells find a suitable environment, they usually stop migrating and begin to form more permanent adhesions. The initial attachment is similar to migration, but then integrin‐based clusters of nascent adhesions develop into stronger adhesions like focal adhesions or even more permanent fibrillar adhesions (**Figure** **3**).^[^ ^47^, ^48^, ^49^ ^]^ This process can be triggered or strengthened in two ways: by adhesive ECM proteins or by activating integrin affinity and clustering through soluble signaling molecules such as growth factors. These two mechanisms often work together, but to different extents.^[^ ^50^ ^]^

#### 2.2.2 Attachment to the Basement Membrane and Formation of Hemidesmosomes

The basement membrane forms the structural foundation of many tissues and consists of the basal lamina, which lies adjacent to cells and contains various ECM glycoproteins such as collagen, laminin, and fibronectin.^[^ ^51^ ^]^ Beneath this, the reticular lamina contains fibrillar collagens produced by fibroblasts in the underlying connective tissue. Alongside typical focal adhesions, cells have another type of longer‐lasting attachment structure, unique to basement membrane attachment, hemidesmosomes (Figure 3).^[^ ^50^ ^]^ These are strong cell‐surface attachments that link the basement membrane to the cell's intermediate filament network, as the filaments bind to the cytosolic part of the hemidesmosome. The primary adhesion receptor is integrin α6β4, which binds laminin in the basement membrane. These integrins are connected to intermediate filaments through intracellular plaques.^[^ ^52^, ^53^ ^]^

### 2.3 ECM Composition

Besides cell–cell contacts influencing cellular behavior, another major factor in migration, adhesion, and overall tissue function is regulated by signals present in the ECM and its structural proteins.^[^ ^21^ ^]^ These protein complexes contain many linear and conformational binding motifs that cell membrane receptors recognize and bind to, thereby initiating adhesion.^[^ ^20^, ^24^ ^]^ More complex interactions may require precise steric conformations to fit into single or clustered receptor binding sites.^[^ ^54^ ^]^ These structural proteins contribute to the ECM's inherent bioactivity and its ability to guide cellular behavior, while also modulating the tissue's mechanical properties. Therefore, we will examine the most common ECM proteins and their functions as ECM components (**Table** **1**).

**Table 1:** The most common ECM structural proteins, their different subtypes, the type of structures they assemble into in the ECM and their function, as well as the tissues they are most abundant in.

ECM Proteins	Types	Formed ECM structures	Function	Tissues
Collagens	28 types (Collagen I‐XXVIII)^[^ ^60^ ^]^	Collagen I most abundant, stiff fibrillar structures/arrangements for type I, II, III, V, XI (50 nm–300 µm diameter). Some subtypes are also non fibrillar or fibril associated.^[^ ^27^ ^]^	Mechanical reinforcement of ECM, stiffening of tissues, cell signaling/attachment.^[^ ^27^ ^]^	Most tissues except brain, most abundant in skin, tendon, and bone.^[^ ^27^, ^55^ ^]^
Elastins	11 isoforms of the main component tropoelastin^[^ ^59^ ^]^	Stretchable fibrils (up to 1 µm diameter).^[^ ^60^ ^]^	Facilitating tissue elasticity, cell adhesion/signaling.^[^ ^59^, ^60^ ^]^	Stretchable tissues; skin, lung, tendon, ligaments.^[^ ^59^, ^60^ ^]^
Fibronectins	2 types, ECM insoluble fibronectin and soluble blood fibronectin^[^ ^70^ ^]^	Fibrillar in its functional form (up to several µm diameter), also has soluble forms in blood plasma.^[^ ^63^, ^70^ ^]^	“Provisional matrix”, binds many ECM proteins and cell‐attractive factors, used to regulate cells depending on its state.^[^ ^63^ ^]^	Embryo, regenerating tissues, soluble form in blood plasma.^[^ ^63^ ^]^
Laminins	16 types^[^ ^67^ ^]^	Form non‐fibrillar mesh‐like structures, most abundant Laminin‐211.^[^ ^71^ ^]^	Essential component of basement membrane, facilitates formation of nerves.^[^ ^67^ ^]^	Basement membranes, endothelium, surrounding neuronal tissue, epithelium, and adipose tissue.^[^ ^67^ ^]^
Tenascins	4 types (TN‐C, TN‐R, TN‐X, TN‐W)^[^ ^68^ ^]^	Non‐fibrillar structures.^[^ ^68^ ^]^	Essential for correct tissue maturation, weak cell adhesion.^[^ ^68^ ^]^	Early tissue development, especially neuronal tissue development, wounds, and tumors.^[^ ^72^ ^]^

**Collagen** is the main structural protein of the ECM and is found in most tissues except the brain.^[^ ^55^ ^]^ Different isoforms can form fibrillar or nonfibrillar structures (Table 1), depending on the type and assembly of the constituent collagen chains.^[^ ^27^, ^56^ ^]^ The most common is type I collagen – a fibrillar, heteromeric complex of three collagen chains forming a triple‐helical structure. These helices assemble into fibrils, which further organize into higher‐order fibers and bundles, crosslinked to provide tissue stiffness and mechanical strength.^[^ ^24^, ^57^ ^]^ Collagen also contains binding motifs for ECM proteins (e.g., vitronectin, fibronectin) and growth factors, and offers binding sites for at least six receptor groups: integrins, receptor tyrosine kinases (Discoidin Domain Receptor), glycoprotein VI, OSCAR, LAIR‐1, and uPARAP/Endo180.^[^ ^58^ ^]^ The specific receptor interactions, along with local growth factors and signals, elicit diverse cellular responses, demonstrating the complexity of ECM–cell interactions.

**Elastin** is responsible for tissue elasticity, allowing tissues to respond to dynamic mechanical stress without permanent deformation,^[^ ^59^ ^]^ which is essential in stretchable tissues.^[^ ^24^, ^60^ ^]^ Additionally, elastin modulates cell signaling by regulating cell proliferation, promoting adhesion, and acting as a chemotactic agent.^[^ ^61^ ^]^

**Fibronectin** is another fibrillar structural glycoprotein (Table 1) that plays a vital role in mediating interactions between cells and the ECM. It is recognized by integrin receptors on the cell membrane and can also bind many other matrix components such as collagens and proteoglycans,^[^ ^62^ ^]^ acting as a linker between these components.^[^ ^63^, ^64^ ^]^ Fibronectin is the protein in which the cell‐adhesive RGD amino acid motif was first discovered and characterized.^[^ ^65^ ^]^ Additionally, fibronectin exists in a soluble form found in blood plasma.^[^ ^66^ ^]^

**Laminins** play a more specific role in mediating cell adhesion to the basement membrane, which is essential in tissues such as the endothelium, surrounding neuronal tissue, epithelium, and adipose tissue.^[^ ^67^ ^]^ Different laminin isoforms influence cell adhesion and migration by providing distinct cell–ECM interactions that are independent of collagen and fibronectin.^[^ ^24^, ^67^ ^]^

**Tenascins** stand out for their highly specific role in early tissue development and their prominent presence in wound and tumor environments.^[^ ^68^ ^]^ During the embryonic phase, they ensure proper development of the nervous system by supporting neuronal and axon growth. Tenascins tend to bind fibronectin, integrins, proteoglycans, and immunoglobulin‐type receptors on cells, which can either inhibit or promote cell adhesion.^[^ ^69^ ^]^

The interaction between cells and ECM components is mutually dependent, shaping the mechanical properties of tissues and influencing cell behavior, including migration.^[^ ^73^ ^]^ During migration, cytoskeletal contractions generate forces that cells use to sense ECM stiffness and adjust accordingly.^[^ ^74^ ^]^ This process, known as durotaxis,^[^ ^75^ ^]^ leads cells to migrate toward regions with stiffness similar to their native environment when a gradient is present.^[^ ^76^ ^]^ Besides stiffness, matrix porosity and degradability also affect migration; these can hinder movement or trap cells. Matrix plasticity, the extent to which the matrix yields to cellular forces, determines whether cells can deform or break ECM bonds to move through it.^[^ ^77^ ^]^ At the tissue level, mechanical stimulation can drive migration, proliferation, and differentiation.^[^ ^78^, ^79^ ^]^ For example, muscle cells require excitation and contraction during culture,^[^ ^80^ ^]^ while cartilage development benefits from compression.^[^ ^81^ ^]^ Dynamic mechanical cues also enhance fluid exchange, improving nutrient delivery and supporting signaling gradient formation.

## 3 Fibrous Extracellular Microenvironments

As outlined earlier, the regulation of cell attachment, proliferation, and migration is vital for both ECM integrity and cell function. Cells dynamically remodel the ECM by degrading, rearranging, or replacing its components, guiding other cells,^[^ ^82^ ^]^ influencing cell fate,^[^ ^83^ ^]^ and supporting repair in wound healing^[^ ^84^ ^]^ or age‐related tissue damage.^[^ ^85^, ^86^ ^]^ Disruption of these interactions can lead to uncontrolled proliferation and migration, which are hallmarks of cancer.^[^ ^87^ ^]^ In tissue engineering, cell densities are typically low during the early stages of culture, giving cells ample space to proliferate and migrate, which amplifies their responsiveness to substrate properties. For example, fibronectin patterns can be applied to significantly affect cell polarization and migration, with aligned patterns increasing speed and directionality as compared to random patterns.^[^ ^88^ ^]^ Though conducted on artificial, non‐fibrous substrates, the same principle also holds for collagen‐based matrices.^[^ ^89^, ^90^ ^]^ Moreover, it is also important to consider cell‐cell interactions that may require a specific initial density, supporting cell types, or defined spatial arrangements. Such factors can strongly influence how cells respond to the ECM. A key example is contact inhibition of locomotion, where cells alter their movement upon encountering another cell.^[^ ^91^ ^]^ Although well‐studied in 2D systems, this behavior can differ in more complex environments. Singh et al.^[^ ^92^ ^]^ explored this by placing two fibroblast cells on either a single fiber or across multiple fibers. They found that cell–cell interactions differed from those observed in 2D settings: on a single fiber, cells often moved past each other after contact, while on multiple fibers, less elongated cells were more likely to realign and migrate together. The balance between cell–cell and cell–substrate affinity is critical. When cells prefer interacting with each other over the substrate, they tend to form aggregates like spheroids,^[^ ^22^ ^]^ organoids^[^ ^93^ ^]^ or cell sheets.^[^ ^94^ ^]^ While this behavior can be advantageous for certain biofabrication strategies, it may pose challenges in other contexts. For example, cells might initially form a stable monolayer but later detach, which is undesirable in experiments that involve scaffold handling or mechanical stimulation.

While 2D or 2.5D substrates already involve complex interactions, this complexity increases in 3D, where cells interact with the matrix and other cells in all directions, and mechanical properties like ECM stiffness also play a role.^[^ ^83^ ^]^ Given this, the following sections will first examine general cell interactions with fibrous substrates, then break down key fiber design parameters such as surface chemistry, topography, diameter, and alignment that can be systematically varied during fabrication. This will be followed by an overview of 3D matrices, including fiber‐reinforced hydrogels and fiber fragment bioinks, summarizing the current state of the art in this area. Understanding both aspects is essential for designing microenvironments that effectively guide cell behavior and tissue development.

### 3.1 Impact of Fiber Properties on Cellular Responses

In tissue engineering and biofabrication, ECM analogs are often fabricated using fiber‐based scaffolds, produced mainly by electrospinning^[^ ^95^ ^]^ or melt electrowriting.^[^ ^96^ ^]^ These are then used as fiber matrices in the form of mats or scaffolds of different geometries, which can also be supplemented or added to a hydrogel.^[^ ^97^ ^]^ The fibrous nature of these scaffolds is intentional, as nano‐ and microfibers mimic structures that would otherwise be composed of fibrillar ECM proteins such as collagen, or larger physiological structures like axons and capillaries. Consequently, these scaffolds can be used to influence cellular behavior, morphology, and overall viability.^[^ ^98^ ^]^ Fibrous scaffolds have been shown to be essential in many cases, as they provide cells with a surface to attach to, guide them, form a basis for their migration, shape, and directionality, help facilitate a 3D environment, and, in part, provide mechanical and topographic cues. They simultaneously stabilize the substrate during maturation and handling.^[^ ^99^ ^]^ The efficacy of such fibrous scaffolds in recapitulating their intended biomimetic role is, nonetheless, much more difficult and nuanced than perhaps initially expected. This is because the cellular response to fibers depends on many factors and often varies depending on the cell type.^[^ ^100^, ^101^ ^]^ The choice of scaffold material is also very important to consider in terms of its biocompatibility and degradability when exposed to cells or bodily fluids.^[^ ^102^ ^]^

#### 3.1.1 Contact Guidance

The cellular response to a substrate or fiber is defined by the interplay of key interaction principles and mechanisms. These generate an integrated response based on which interactions predominate under given circumstances. The most common principle is contact guidance, where cells repolarize in response to topographic features. While the precise molecular mechanisms remain unclear, the focus is on the cellular responses when cells interact with these features. Contact guidance is influenced by chemical cues (e.g., surface chemistry or signaling gradients), mechanical cues (e.g., matrix stiffness), and steric features (e.g., porosity, fiber size, and shape).^[^ ^103^ ^]^ These cues induce polarization and directed migration as cells follow chemical or topographic gradients. A simple example is seen in aligned structures like ECM protein lines, grooves, or fibers, where cells align and migrate along them. Here, cell shape and protrusions are polarized by myosin II contractility along non‐adherent edges, suppressing protrusion activity. Meanwhile, positive feedback at focal adhesion sites increases binding avidity, leading to greater polarization and formation of leading edge protrusions, dependent on local lamellipodia orientation and size.^[^ ^104^, ^105^ ^]^

#### 3.1.2 Curvotaxis

Another important interaction principle is curvotaxis, where some cells migrate toward concave regions relative to their size. This behavior is partly driven by the shape and mechanical constraints of the nucleus, as cells prefer to maintain a spherical, less compressed nucleus. As a result, cells migrate into concave regions rather than across convex surfaces, and the nucleus may even reposition itself.^[^ ^106^ ^]^ This process is regulated by interactions between the cytoskeleton and nucleoskeleton, and can influence gene expression based on nuclear shape.^[^ ^106^, ^107^ ^]^ When applied to fibers with diameters similar to or larger than the cells, cells tend to elongate along the fiber axis, reducing nuclear compression.

**Figure 4:**
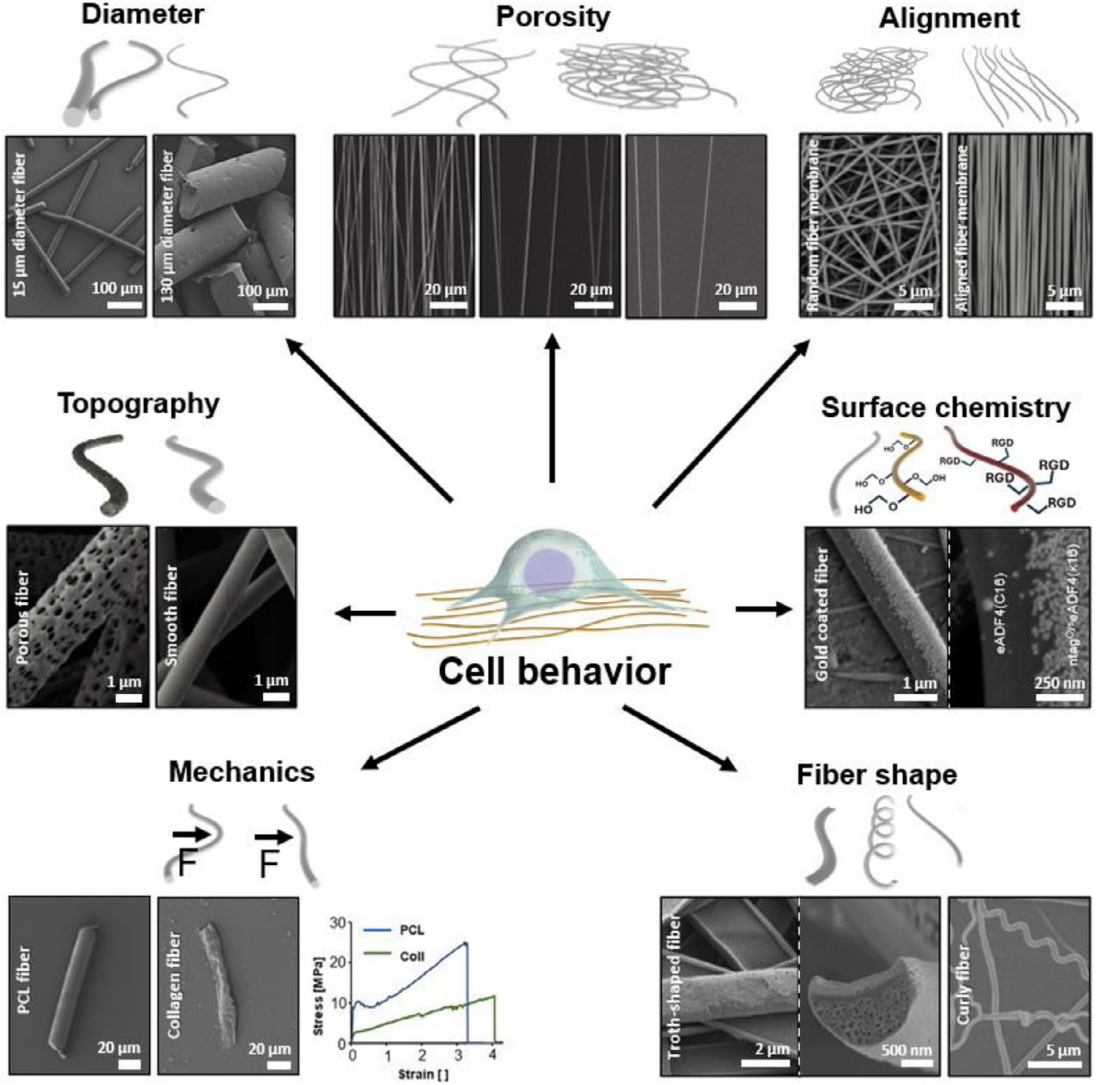
Characteristics of fibers that influence cell behavior. One influential factor is the different fiber diameters, with examples of 15 and 130 µm diameter cut fibers shown. Reproduced from Ref. [109] Copyright (2024), with permission from Wiley‐VCH GmbH. The macro‐porosity/fiber‐fiber distance of a substrate is another influence; this is demonstrated with aligned electrospun substrates with different‐sized pores/gaps. Reproduced from Ref. [110] Copyright (2011), with permission from Wiley‐VCH GmbH. The alignment of these fibers can also vary, as shown with a random and aligned electrospun membrane. Reproduced from Ref. [111] Copyright (2021), with permission from Springer Nature. Fibers may also have a different surface chemistry, as an example a side‐by‐side fiber made of two recombinant spider silk variants, with one later covalently coupled to gold particles. Reproduced from Ref. [112] Copyright (2022), with permission from Wiley‐VCH GmbH. As an example for different fiber shapes, a troth shaped and curly fiber are shown. Reproduced from Ref. [113, 114] Copyright (2020), with permission from the American Chemical Society, and Copyright (2022), with permission from MDPI respectively. The different mechanical properties that fibers may have, here represented by stiff polycaprolactone (PCL)P fibers compared to softer collagen I fibers, with the corresponding stress strain diagram. Reproduced from Ref. [115] Copyright (2024), with permission from Wiley‐VCH GmbH. Surface topography is another alternative to vary the fibers characteristics, here exemplified by porous and smooth PLA fibers. Reproduced from Ref. [116] Copyright (2025), with permission from Springer Nature.

Cells typically respond to multiple adhesive and topographical cues simultaneously, which often interact in complex ways. Because different cell types perceive and integrate these signals differently, general design guidelines should be applied with caution and specific responses should be pre‐screened whenever possible.^[^ ^108^ ^]^ Experiments that aim to isolate the effect of topography alone are challenging, as changes in geometry also influence factors such as cell distribution, substrate stiffness, and diffusion. Ultimately, it is the interplay between chemical and topographical cues that defines cellular behavior on engineered scaffolds. Based on these principles, fibrous scaffold design must address both micro‐ and macroscale parameters. Microscale aspects include surface chemistry and topography, while macroscale features such as fiber diameter, spacing, shape, alignment, and mechanics are equally crucial for guiding cell organization and tissue formation. (**Figure** **4**).

#### 3.1.3 Fiber Surface Chemistry

The performance of fibrous and non‐fibrous substrates depends on a range of micro‐ and macroscale factors and their complex interplay. At the molecular level, the surface chemistry, which describes the specific molecules present at the material's surface, is a key determinant of cellular responses. These surface molecules interact directly with the cell membrane and its receptors, modulating cell adhesion, which can be either enhanced or inhibited depending on the chemical composition and the functional groups exposed.^[^ ^117^ ^]^ Regardless of whether flat, well‐like surfaces or more complex geometries, such as fibers, are used, surface chemistry remains a critical factor that must be considered. It is one of the main determinants of effective cell attachment and proliferation and can, if inadequate, outweigh the benefits of any macroscopic design considerations. Molecules commonly used to promote cell adhesion are typically derivatives of ECM proteins,^[^ ^24^ ^]^ such as collagen, laminin, gelatin, shorter peptide sequences, or engineered protein constructs carrying specific tags known to enhance cell‐mediated adhesion, most notably the RGD sequence, which facilitates binding to integrin receptors.^[^ ^65^ ^]^ As discussed in the chapters on cell adhesion and migration, the activation and clustering of these receptors into larger complexes, in conjunction with the cytoskeleton, are essential for effective cell adhesion and migration. To enhance adhesion, cell‐adhesive proteins or peptides can be chemically bound or physically adsorbed onto the substrate surface.^[^ ^118^, ^119^, ^120^, ^121^ ^]^ Importantly, even without prior chemical modification, the innate hydrophobicity or hydrophilicity of a material significantly influences protein adsorption from the surrounding medium. This plays a crucial role, as surfaces with a higher affinity for cell‐attractive proteins can promote better cell binding, depending on the nature and composition of the adsorbed proteins.^[^ ^122^ ^]^

The importance of tailoring surface chemistry becomes particularly evident when considering the opposite case when a material is inherently unattractive to cells. This is commonly achieved through the use of anti‐fouling materials that are hydrophilic and exhibit low protein adsorption, thereby preventing the presentation of cell‐adhesive molecules. In such cases, the underlying topography becomes irrelevant: if the surface is inert to cell–receptor interactions, cells will neither adhere nor migrate. This represents a critical limitation for materials intended to support cellular attachment and proliferation. Examples of such cell‐repellent surfaces include coatings composed of polyethylene glycol (PEG), polyethylene oxide (PEO), or dextrans, which are materials known for their high hydrophilicity and lack of biochemical affinity for cell adhesion.^[^ ^123^, ^124^ ^]^ Similarly, surfaces coated with non‐adhesive proteins such as bovine serum albumin (BSA) are often employed to minimize cell interaction.^[^ ^124^ ^]^ Another notable example is recombinant spider silk, particularly the unmodified variant eADF4(C16), which has been established as a cell‐repellent and antifouling substrate across various morphological formats.^[^ ^125^, ^126^, ^127^, ^128^ ^]^

The impact of surface‐bound chemical cues in mediating cellular attachment was demonstrated by Lamberger et al.,^[^ ^129^ ^]^ who employed naturally cell‐repellent recombinant spider silk microarrays,^[^ ^130^ ^]^ which became highly adhesive to cells when modified with an RGD motif. Adding cell capturing aptamers to the unmodified arrays similarly enabled cell capture and eventual spreading, even without innate adhesive queues. Highlighting surface chemistry's crucial role in cell adhesion and retention on the material.

A similar influence of surface chemistry is observed in fibrous materials composed of hydrophobic polymers or polymer blends. These materials tend to adsorb a higher amount of cell‐attractive proteins from the surrounding medium, thereby enhancing cellular adhesion and proliferation. A particularly illustrative example was provided by Su et al.,^[^ ^131^ ^]^ who fabricated side‐by‐side fibers composed of pure PCL and a blend of PCL with PEO. The cells showed a clear preference for adhering to the side of the fiber with lower PEO content, indicating reduced cell and protein adhesion on the more hydrophilic component.

This highlights the importance of carefully selecting polymers based on their hydrophobic or hydrophilic characteristics, as this can strongly influence cellular interactions. Nevertheless, even intrinsically non‐adhesive surfaces can be rendered bioactive through chemical modification—for example, by coating them with adhesion‐promoting peptides or extracellular matrix proteins. ^[^ ^122^, ^132^, ^133^ ^]^ However, it is important to note that the effectiveness of such surface functionalization may still depend on the physicochemical properties of the underlying polymer.

Modulating surface chemistry offers a powerful means of influencing cellular signaling. The specific molecules, or combinations thereof, present at the interface can be designed to fine‐tune cellular responses. For example, functionalizing surfaces with ligands that activate membrane receptors, such as epidermal growth factor (EGF), has been shown to enhance processes like cell adhesion and migration.^[^ ^134^ ^]^

In more advanced applications, the same signaling molecules, or synergistic mixtures, can be arranged to form directional gradients, further guiding cellular behavior. These spatiotemporal patterns, whether through immobilized cues or soluble factors, can direct cell fate decisions and migration pathways. Such strategies aim to mimic the dynamic and spatially regulated signaling environments characteristic of tissue development, effectively substituting for hormonal or paracrine stimuli that are not directly mediated by the extracellular matrix.^[^ ^135^, ^136^ ^]^

#### 3.1.4 Fiber Topography

Another important factor influencing cell adhesion is the nano‐ and microscale topography of the substrate. In its simplest form, this refers to increased surface roughness, but it can also include more complex, patterned structures. Rough surfaces offer a larger contact area, allowing more opportunities for cellular attachment and for adhesive molecules to accumulate. Additionally, indentations, grooves, or microstructures such as pillars can provide physical cues that improve anchoring or promote specific cell behaviors. These effects are not limited to flat substrates but also apply to fibrous materials. Porous fibers, for example those made from chitosan,^[^ ^137^ ^]^ have been shown to enhance cell adhesion. In some cases, higher surface roughness has also been associated with increased cell migration. Lampin et al.^[^ ^138^ ^]^ observed that vascular and corneal cells exhibited greater migration rates on sandblasted poly(methyl methacrylate) (PMMA) surfaces compared to smoother counterparts. It should be noted, however, that excessive surface roughness or porosity can produce features that are relatively large compared to the cells, which might induce undesired effects.^[^ ^139^ ^]^ Cells may spread poorly, adopt irregular shapes, or exhibit altered functionality and alignment, which can be detrimental depending on the intended application.

The impact of surface topography on cell adhesion and directional behavior was clearly demonstrated by Ryma et al.,^[^ ^140^ ^]^ who developed a melt electrowriting technique to produce PCL fibers composed of aligned nanofiber bundles. These bundles followed the direction of fiber deposition and provided a structured surface that significantly enhanced cell attachment compared to smooth fiber controls. Furthermore, the aligned nanofibers promoted elongation and polarization of macrophage cells along the fiber axis, resulting in guided migration along the same direction.

There are numerous approaches to structuring the surface of a substrate, whether flat or fibrous. However, the transferability of these techniques often depends on the material and fabrication method used.^[^ ^141^ ^]^ When designing surface features, it is also essential to consider the type of cells being cultured.

#### 3.1.5 Fiber Diameters

Similar to the influence of surface structuring, the diameter of individual fibers also plays a significant role in modulating cellular behavior. In fibrous mats or scaffold systems, varying the fiber diameter alone can lead to pronounced differences in cell response. Importantly, changing the diameter also affects key substrate properties, such as porosity and the spacing between fibers.^[^ ^142^ ^]^ To isolate the effect of fiber diameter, experiments are ideally conducted using single fibers or configurations in which the distance between fibers is sufficiently large to prevent interactions with neighboring structures. Otherwise, confounding effects from fiber alignment or density can mask the influence of diameter. There are three main cellular behaviors known to be affected by fiber diameter: polarization along the fiber axis, adhesion strength, and migration rate.^[^ ^143^, ^144^ ^]^ While responses vary depending on cell type, a general trend can be observed. Micrometer‐scale fibers tend to promote cell elongation and polarization, whereas nanofibers typically enhance adhesion.^[^ ^11^, ^145^, ^146^ ^]^ However, when the fiber diameter significantly exceeds that of the cell, the cell's ability to sense the curvature of the fiber diminishes. As a result, polarization and guided migration along the fiber are reduced.^[^ ^147^ ^]^ Conversely, if the diameter becomes too small, the fiber may no longer provide sufficient topographic cues for alignment. Thus, there appears to be an optimal diameter range in which cells can most effectively sense and respond to the fiber's geometry, with both lower and upper limits beyond which alignment and function are diminished.

For specific cell types and populations, there is a growing body of literature that more precisely addresses fiber diameter preferences. It is important to distinguish between the initial phases of attachment and polarization, and the later development of mature focal adhesions.^[^ ^148^, ^149^ ^]^ In scaffold design, this distinction becomes relevant when deciding whether the primary goal is to promote cell migration or stable attachment, as enhanced migration may come at the expense of strong adhesion and long‐term proliferation.

When fibers are arranged in close proximity and aligned, diameter plays an additional role in modulating cell behavior. Smaller‐diameter fibers collectively offer a greater surface area for adhesion and typically form narrower inter‐fiber grooves.^[^ ^146^ ^]^ This tends to promote stronger attachment. In contrast, larger fibers on the scale of a few micrometers are more effective in guiding cell alignment, as their topography is more readily perceived by cells.^[^ ^145^ ^]^ Thus, adhesion strength and polarization may at times represent competing outcomes, requiring a compromise in scaffold design. This trade‐off was demonstrated by Łopianiak et al.^[^ ^150^ ^]^ who showed that endothelial cells adhered more strongly to nanofibers, likely due to the increased available surface area, but displayed greater elongation and alignment on microfibers.^[^ ^145^, ^146^ ^]^ Once again, these findings highlight the critical role of topography and surface area in supporting both attachment and directional guidance of cellular behavior.

The diameter of the fibers also influences the mechanical properties of the scaffold and its interaction with the surrounding environment. This becomes particularly relevant in applications involving dynamic fluid flow, such as in vascular systems. In such cases, the effect of fiber size on fluid dynamics must be carefully evaluated. Larger fiber diameters have been associated with the induction of turbulence and an increased risk of blood coagulation.^[^ ^151^ ^]^ Therefore, a thorough assessment of fiber diameter, geometry, and mechanical behavior is essential to ensure functional compatibility in flow‐exposed environments.^[^ ^152^, ^153^ ^]^

Beyond their role in modulating attachment, alignment, and migration, fiber diameters can also influence cellular gene expression. In some cases, fiber size has been shown to affect the differentiation pathways of stem or progenitor cells.^[^ ^121^ ^]^ For example, Gu et al.^[^ ^147^ ^]^ demonstrated that rat bone marrow mesenchymal stem cells exhibited lower osteogenic differentiation potential on fibers with a diameter of 1 µm compared to those cultured on larger fibers of 8 and 56 µm. These findings suggest that fiber diameter is not only a structural parameter but also a functional cue capable of influencing cell fate.

When considering the long‐term behavior of scaffold–cell systems, another important parameter that can be influenced by fiber diameter – beyond immediate cell interaction – is the degradability of the scaffold.^[^ ^154^ ^]^ While the choice of material is the primary determinant for degradation kinetics, the degradation rate can also be modulated through structural features. For example, incorporating pores or other surface modifications that increase surface area and enzymatic accessibility can significantly accelerate breakdown.^[^ ^137^ ^]^ Interestingly, many of these degradability‐enhancing features are also favorable for cellular attachment and proliferation, provided they are appropriately scaled. In addition to commonly used biodegradable polymers such as polylactic acid (PLA), PCL, and polydioxanone (PDO), materials based on gelatin, collagen, or silk have also been employed to achieve tunable degradation profiles, ^[^ ^155^ ^]^ particularly in applications such as wound healing.^[^ ^156^ ^]^

#### 3.1.6 Fiber Alignment

Fibrous substrates introduce an additional level of topographical cues that cells can perceive: the degree of fiber alignment. This structural characteristic also has implications for the scaffold's pore size, which is influenced not only by the arrangement of the fibers but also by their diameter.^[^ ^157^ ^]^ Larger fiber diameters inherently lead to larger pores and wider inter‐fiber gaps, affecting both cell behavior and scaffold porosity. Because cell migration is not limited to individual fibers – especially when fibers are closely spaced – cells extend filopodia to probe multiple neighboring fibers while typically migrating along a dominant fiber. This behavior can shift rapidly depending on fiber proximity, angular orientation, and diameter. In the case of aligned submicrometer fibers, fiber density becomes a key parameter, as it defines the spacing between fibers and, consequently, the distance a cell must bridge to contact adjacent fibers. Closer spacing facilitates sensing and spreading across multiple fibers.

Park et al.^[^ ^110^ ^]^ demonstrated that beyond a critical fiber–fiber distance, the contact guidance effect is reduced, resulting in diminished cell polarization. When the gaps between fibers exceed the size of the cell, cells tend to spread across multiple directions rather than aligning along the fiber axis. Optimal polarization was observed when fiber spacing remained below the typical cell diameter. Furthermore, Gu et al.^[^ ^147^ ^]^ showed that fiber density played a crucial role in the osteogenic differentiation of stem cells on aligned PCL fiber matrices. In their study, denser fiber arrangements enhanced differentiation outcomes, highlighting that both fiber diameter and spacing must be considered together in scaffold design.

In addition to fiber diameter and spacing, the degree of fiber alignment, defined by the angles between individual fibers, also plays a key role in regulating cell shape, migration trajectories, and migration speed. Li et al.^[^ ^145^ ^]^ demonstrated this interplay using endothelial cells, showing that while cells preferentially adhered to nanofibers, they exhibited better alignment on microfibers. However, when the same fibers were arranged in random orientations rather than aligned patterns, cellular polarization decreased. This was attributed to the lack of consistent directional cues resulting from non‐uniform fiber angles. Further evidence was provided by Wang et al.,^[^ ^158^ ^]^ who investigated the migratory behavior of KGN tumor cells and HEK 293T cells on nanofibrous scaffolds with varying degrees of fiber alignment. Their study found that KGN cells, in particular, showed pronounced sensitivity to fiber orientation, with marked differences in velocity, migration trajectories, and cellular morphology depending on the alignment of the underlying scaffold.

The development of advanced techniques such as melt electrowriting has enabled the fabrication of increasingly complex and precisely controlled fiber architectures. This allows scaffolds to be tailored to match specific cellular preferences and to promote more physiologically relevant behavior. As a result, cells can adopt morphologies and functions that more closely resemble their native environment, rather than appearing deformed or functionally impaired.^[^ ^100^, ^159^ ^]^

When evaluating the overall behavior of cells within a scaffold, it is important to consider that changes in fiber spacing, diameter, porosity, or material composition inherently alter the mechanical properties of the construct. This adds a further layer of complexity to the already intricate interplay of physical and biochemical cues. Mechanical factors are particularly relevant for mechanosensitive cells, which respond to substrate stiffness or migrate along stiffness gradients. Some cell types, such as osteoblasts, favor stiffer environments similar to the native bone ECM, whereas others, such as neurons, require softer substrates to function properly.^[^ ^160^ ^]^ It is essential to distinguish between the mechanical properties of the scaffold as a whole and those that are locally sensed by cells. Cells typically respond only to mechanical signals within their immediate microenvironment, interacting with fibers through localized traction and focal adhesion formation. In contrast, the macroscopic mechanical behavior of the scaffold becomes more relevant at later stages, especially when the engineered tissue must withstand mechanical loading or integrate with host tissue. Given the complexity of this topic, we refer to existing comprehensive reviews for further detail.^[^ ^161^, ^162^ ^]^ Nonetheless, mechanical properties represent an important design parameter in fibrous scaffolds and must be considered in parallel with structural and biochemical factors.

A thorough understanding of the interplay between fiber alignment, spacing, diameter, and the resulting mechanical properties has important practical implications. Such parameters can be specifically tailored to direct cellular behavior in applications such as wound healing,^[^ ^163^ ^]^ engineering of aligned tissues including muscle, neuronal tissue,^[^ ^164^ ^]^ and blood vessels,^[^ ^146^ ^]^ or in cancer research. In the tumor microenvironment, aligned collagen fiber architectures are known to influence cancer cell behavior, playing a role in processes such as tumor progression and metastasis.^[^ ^90^ ^]^ Fibrous scaffolds are also highly valuable in experimental settings. Due to their 3D architecture, increased surface area, and improved ability to guide cells, thick porous electrospun membranes and melt electrowritten scaffolds that cells can infiltrate, often outperform traditional 2D culture substrates in supporting cell proliferation and organization. Furthermore, they better mimic the natural extracellular environment of many tissues, thereby inducing more physiologically relevant cellular behavior. This is particularly important for applications in drug testing and disease modeling.^[^ ^165^ ^]^ Additionally, mass transport within fibrous scaffolds can be enhanced when they are suspended in bioreactors that enable perfusion from both above and below the cell layers. Such systems improve nutrient and gas exchange and further support the development of functional tissue constructs.^[^ ^166^ ^]^

#### 3.1.7 General Guideline for Fiber Design

When the dimensions of the fibers are smaller than those of the cell, they are more likely to act as topographical features that influence adhesion and migration. In such cases, the mechanical cues introduced by fiber geometry are more readily sensed by the cells at the microscale. Conversely, when fiber dimensions exceed cell size, the overall shape of the fiber can strongly influence cell guidance. Under these conditions, cells may attempt to adapt their morphology and migration patterns to the geometry of the surrounding fibers to a certain extent. Overall, the influence of fiber characteristics on cellular behavior is highly complex and strongly dependent on the specific cell type. Determining the optimal combination of fiber parameters for a desired biological outcome is therefore a challenging task. Nonetheless, some general trends and recommendations can be derived to anticipate cellular responses (**Table** **2**). In addition to size and alignment, the shape of individual fibers can also be modified. Curved,^[^ ^167^ ^]^ hollow,^[^ ^168^ ^]^ or helical^[^ ^114^, ^131^ ^]^ fibers are examples of such variations. While predicting the exact effects of these geometries on cell behavior remains challenging, certain trends can be inferred based on prior observations.

**Table 2:** A general guideline of how fiber characteristics influence cellular behavior.

Fiber characteristic	Features	Induction of cellular response
Chemistry	Cell attractive molecules/cues:	Essential for modulating cell adhesion.^[^ ^24^, ^118^ ^]^
	Surface wettability:	Influences adsorption of cell attractive/unattractive proteins.^[^ ^131^ ^]^
	Chemical composition:	Important to be aware of potential cytotoxicity of material, or any other potential influences on cells like radicals, ions, pH or charges.^[^ ^102^, ^169^–^171^ ^]^
Topography	Small features compared to cell size:	Tendentially improve adhesion, may influence cell polarization.^[^ ^137^, ^138^, ^140^ ^]^
	Cell sized or even larger features:	Decreasing impact on adhesion and more likely to alter the cell morphology to that of the features.^[^ ^139^ ^]^
Diameter	Nanofibers:	Improve adhesion, if aligned polarize the cells.^[^ ^145^, ^146^, ^150^ ^]^
	Small microfibers:	Greater polarization along the fiber compared to nanofibers. Decrease in adhesion strength compared to nanofibers.^[^ ^145^, ^146^, ^150^ ^]^
	Larger microfibers:	Fibers much larger than the cells perform worse at polarizing the cells.^[^ ^147^ ^]^
Distance/porosity	Small fiber‐fiber gaps:	Polarization of cells along the fibers in an integrated response to their general direction. Good adhesion.^[^ ^110^ ^]^
	Large fiber‐fiber gap:	Decreasing polarization in fiber direction with increasing gap. Decreasing adhesion.^[^ ^110^ ^]^
	Very large fiber‐fiber gap:	Beyond a large enough gap, cells again start polarizing along a single fiber. Adhesion improves.^[^ ^110^ ^]^
	Low porosity:	Improved adhesion, lower cell infiltration into scaffold.^[^ ^172^ ^]^
	High porosity:	Decreased adhesion, higher infiltration into scaffold.^[^ ^172^ ^]^
Alignment	High alignment:	Cells tend to polarize and migrate along the fiber direction. Higher migration rates.^[^ ^145^, ^158^ ^]^
	Low alignment:	Lower cell polarization, slower migration, no directed migration.^[^ ^145^, ^158^ ^]^
Mechanics	Fiber stiffness:	Cells may prefer different stiffnesses depending on their natural environment, highest relevance is when inducing cell differentiation.^[^ ^160^–^162^ ^]^
	Fiber flexibility:	Flexible/malleable fibers are more prone to deformation by the cells.^[^ ^173^–^176^ ^]^
Fiber shape	Small fiber features:	Cells likely grow over the fiber, greater contribution to adhesion and alignment. Impact of fiber mechanical properties.^[^ ^110^ ^]^
	Large fiber features:	Depending on fiber shape, the cells will likely orient along the fiber, being influenced by its shape.^[^ ^131^ ^]^

### 3.2 Fibers in 3D Scaffolds

In contrast to thin cell patches, barriers, or monolayers, most biological tissues are inherently 3D. Therefore, when aiming to model or reconstruct such tissues, the scaffolds into which cells are seeded must also possess a 3D architecture. This dimensionality is crucial for mimicking native tissue environments, where nutrient and signaling molecule diffusion is limited and cell density is considerably higher.^[^ ^177^, ^178^ ^]^ A 3D scaffold provides greater surface area for cell–cell and cell–matrix interactions, enabling contact from multiple directions. It also allows cells to adopt a more physiologically relevant morphology, in contrast to the flattened, elongated shapes typically observed on 2D substrates. In 2D cultures, cell–surface adhesions dominate, while cell–cell interactions are comparatively reduced.^[^ ^179^ ^]^ The spatial organization provided by 3D environments is essential for replicating in vivo behavior. This is particularly important for applications such as drug screening, where compound efficacy may be significantly compromised in 3D due to reduced diffusion, limited receptor accessibility, and altered cellular response‐factors that have contributed to the failure of many drug candidates.^[^ ^180^, ^181^ ^]^ Furthermore, the functional replication of organ‐level units, such as vascular networks,^[^ ^182^ ^]^ or other complex 3D structures, can only be accurately achieved within a 3D context.^[^ ^183^ ^]^

A commonly used approach to create 3D tissue constructs involves the use of hydrogels. These polymer networks with high water content resemble the native ECM and are typically self‐supporting, allowing cells to be immobilized at different depths from which they can proliferate. In many cases, hydrogels are combined with fiber‐based substrates to improve their mechanical stability, enhance handling, or direct cellular behavior. This strategy mimics natural composite structures such as collagen‐based ECM.^[^ ^184^, ^185^ ^]^

#### 3.2.1 Impact of Fibrous Matrices

While the influence of fiber–cell interactions has been discussed in earlier sections, their role within composite hydrogel–fiber constructs introduces new layers of complexity. In these hybrid systems, cells are exposed not only to fibrous topographies but also to the surrounding hydrogel matrix, whose biochemical and mechanical properties may differ markedly from those of the fibers. This duality can be harnessed to fine‐tune cell behavior, yet it also poses challenges related to material compatibility, cellular accessibility, and structural integration. For example, the cellular response may differ depending on whether the hydrogel is more or less adhesive than the fibers. Similarly, fibers can be deliberately rendered non‐adhesive if only mechanical reinforcement is desired without guiding cell behavior.^[^ ^186^ ^]^ This relationship introduces numerous possibilities for modulating cellular responses through the interplay between fibers and the surrounding hydrogel. In addition to biochemical compatibility, physical properties of hydrogels such as stiffness, porosity, and degradability play crucial roles in influencing cell behavior. Notably, even a highly bioactive electrospun or electrowritten scaffold becomes ineffective if the encapsulating hydrogel prevents cells from migrating, spreading, or contacting the fibers. For a more detailed overview of how hydrogel characteristics affect cellular behavior independently of fibers, several comprehensive reviews are available.^[^ ^187^, ^188^ ^]^ Ultimately, both the fibrous component and the hydrogel matrix must be carefully selected and optimized–not only to achieve the desired individual effects, but also to ensure that their combined interaction supports the intended biological outcome.^[^ ^189^ ^]^

#### 3.2.2 Infiltration of Fibrous Scaffolds

**Figure 5:**
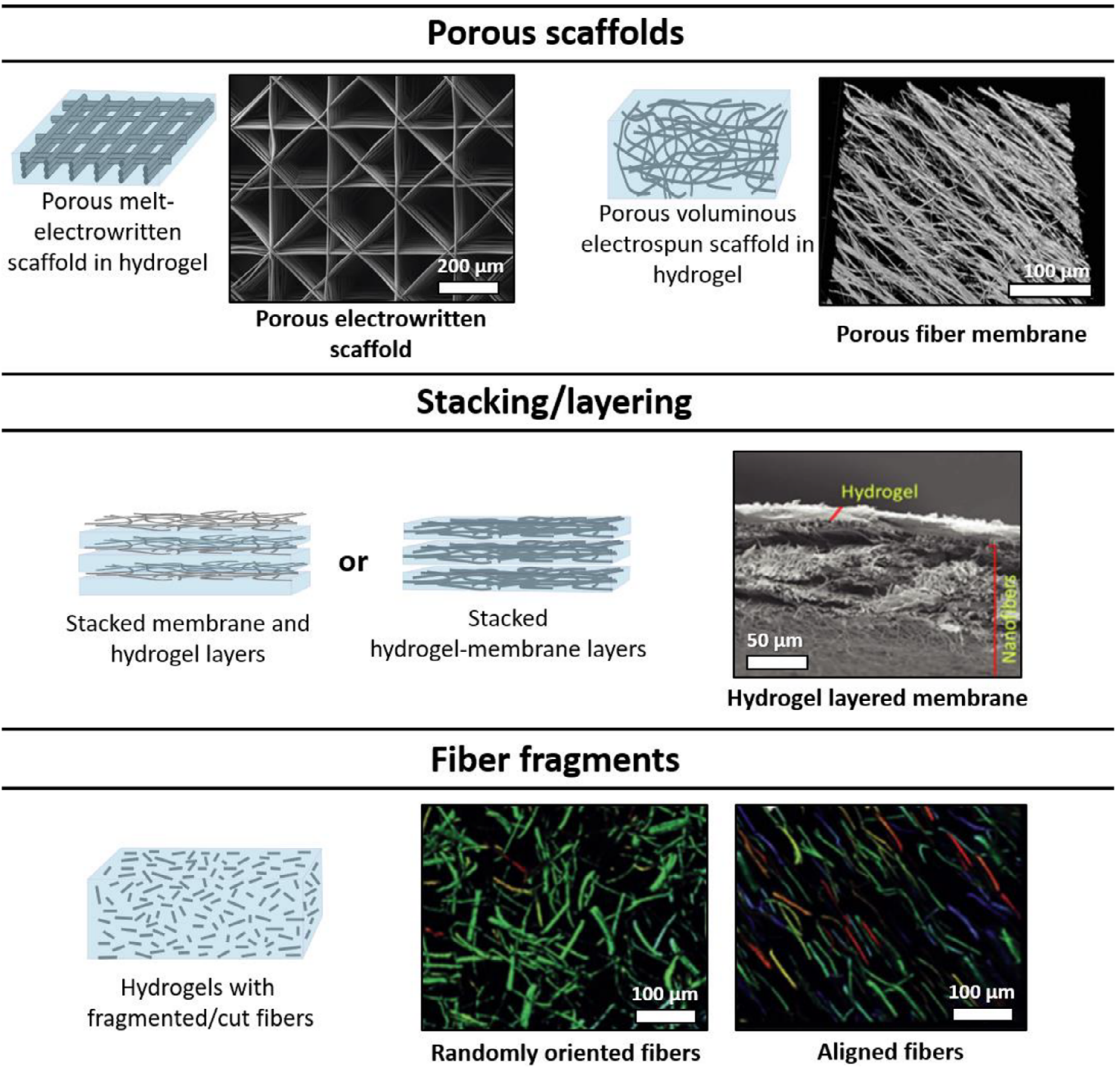
Different approaches of integrating fibers into hydrogels. One alternative is using porous scaffolds, as here exemplified by a porous electrowritten scaffold and a porous PCL fiber membrane Reproduced from Ref. [176, 199] Copyright (2025) with permission from Wiley‐VCH GmbH and Copyright (2022), with permission from with permission from Wiley‐VCH GmbH, respectively. Substrates can also be produced by stacking membrane and hydrogel layers, or layers of membranes already immersed in the hydrogel, as an example an image of a dried nanofiber membrane and hydrogel composite is shown Reproduced from Ref. [211] Copyright (2022), with permission from Springer Nature. The last common alternative of incorporating fibers in hydrogels is by using fiber fragments, here exemplarily shown with magnetic fiber fragments randomly distributed and aligned in a fibrin hydrogel. Reproduced from Ref. [212] Copyright (2017), with permission from Wiley‐VCH GmbH.

There are several strategies for incorporating fibrous structures into hydrogels (**Figure** **5**). The most straightforward approach is to deposit or crosslink a hydrogel directly onto a pre‐formed fibrous scaffold,^[^ ^190^ ^]^ such as an electrospun membrane or a melt electrowritten mesh. This method is also suitable for integrating volumetric scaffolds, including high‐density melt electrowritten grids or electrospun sponges. However, a key limitation of these systems is the extent to which cells or hydrogel precursor solutions can penetrate the fiber network. As shown by Weigel et al.^[^ ^176^ ^]^ salt crystals were used as porogens during electrospinning to create highly porous membranes. This deliberate design significantly enhanced cell infiltration, making the membranes well‐suited for constructing a three‐layer skin model. Beside membrane porosity, cell penetration also depends on factors such as, material wettability, the viscosity of the hydrogel precursor, and the size of the cells.^[^ ^172^ ^]^ In some cases, improved integration may be achieved by techniques such as electrospraying hydrogel–cell mixtures or co‐spinning fibers with hydrogel components.^[^ ^191^ ^]^ Nevertheless, these approaches do not always fully overcome the limitations of scaffold infiltration. An additional consideration is the wettability of the fibrous substrate, which strongly influences the degree of integration between the fibers and the hydrogel matrix. Surface modification strategies, such as coating fibers with hydrogel‐compatible materials or fabricating fibers from the same material as the hydrogel itself, have shown promise in improving interfacial fusion.^[^ ^192^, ^193^ ^]^ Such modifications may also enhance the bioactivity of the scaffold by increasing its attractiveness for cellular adhesion.^[^ ^194^ ^]^

#### 3.2.3 Layer‐by‐Layer Assembly of Fibers and Hydrogels

Another approach for integrating fibrous scaffolds into hydrogels involves stacking multiple fiber layers or alternating them with hydrogel layers.^[^ ^195^ ^]^ In this configuration, the scaffold forms a continuous network within the same plane of the hydrogel but may lack vertical connectivity between layers. This structural organization affects the mechanical properties of the construct and may influence how mechanical stimuli are transmitted and sensed across the system.^[^ ^196^, ^197^ ^]^ Additionally, layered assemblies can be prone to delamination, and the initial cell distribution, alignment, and proliferation rates may vary depending on proximity to the fibrous components. Cells located near electrospun or electrowritten scaffolds are more likely to interact with them, while cells in regions lacking direct fiber contact may behave differently. Therefore, both the layering technique and the hydrogel crosslinking strategy require careful optimization.^[^ ^195^ ^]^ An interesting approach to this layering technique was employed McMahon et al.^[^ ^198^ ^]^ who developed a method in which smooth muscle progenitor cells were first seeded onto an electrospun polyurethane mesh. After two days of cell culture, the mesh was rolled up and combined with a fibrin hydrogel to form a multi‐layered tubular, vessel‐like structure intended for use as a coronary bypass graft.

#### 3.2.4 Fiber Matrices for Structural Support

The reinforcing role of fibrous scaffolds is particularly useful in soft tissue models such as brain or neuronal tissue, where very soft hydrogels are typically used to mimic the mechanical properties of the native environment. These hydrogels often require structural support to be handled and stabilized, which can be achieved through integrated fiber matrices. For example, melt electrowritten scaffolds have been successfully applied in the development of brain tumor models. Andrade Mier et al.^[^ ^199^ ^]^ for instance employed triangular melt‐electrowritten scaffolds to support a soft hydrogel designed to mimic the brain ECM. In this setup, the triangular pore geometry not only provided structural support but also promoted a more native‐like cell morphology, an essential feature for accurately studying neuron–tumor interactions.^[^ ^159^ ^]^ Hence also in 3D, if the fibers are biofunctionalized to be attractive to cells, they can influence behavior through topography, chemical cues, size, shape, and spatial arrangement, similar to their effects in 2D cultures. This can lead to improved proliferation, more physiologically relevant cell morphology,^[^ ^159^ ^]^ and the induction of anisotropic cellular organization.^[^ ^200^ ^]^

#### 3.2.5 Reinforcing Load‐Bearing Hydrogels

Fiber‐reinforced hydrogels are also essential in models of load‐bearing tissues such as ligaments,^[^ ^201^ ^]^ cartilage,^[^ ^202^ ^]^ or meniscal structures. Beyond structural reinforcement, fibers can also be used to stimulate cells via external cues. For example, fibers embedded in hydrogels can serve as conductive elements for electrical stimulation,^[^ ^203^ ^]^ a strategy relevant for muscle,^[^ ^204^, ^205^ ^]^ and neural tissue engineering.^[^ ^206^, ^207^ ^]^ Additionally, in wound healing applications, fiber–hydrogel composites provide mechanical stability, enhance cell viability, and can be functionalized for drug delivery.^[^ ^208^, ^209^ ^]^ Finally, by combining different fiber geometries and scaffold densities, it is possible to more accurately replicate the mechanical behavior and collagen fiber organization of complex tissues. For example, Girard et al.^[^ ^210^ ^]^ developed a composite scaffold by combining electrospun and melt electrowritten layers. Specifically, the electrospun layer was designed to mimic the dermo‐epidermal junction and to limit cell infiltration from the underlying electrowritten structure. The electrowritten layers, representing the dermis, provided the scaffold with its mechanical integrity and played a key role in guiding cell orientation.

#### 3.2.6 Impact of Fibers as Hydrogel Fillers

While the use of entire fibrous membranes or scaffolds is a simple and effective approach for basic tissue models, it is often insufficient for fabricating more complex constructs involving intricate geometries, multiple cell types, or larger volumes. An alternative strategy is to mechanically fragment fibrous scaffolds into smaller fiber segments^[^ ^109^ ^]^ and incorporate them directly into a hydrogel matrix. Unlike intact scaffolds, these short fibers do not entangle or clog during pipetting or extrusion, yet they still provide a fibrous component that reinforces the hydrogel and can be sensed by cells.^[^ ^115^ ^]^ This approach enables the formulation of pipettable or printable bioinks that combine the moldability of hydrogels with the mechanical and topographical benefits of fibrous structures. Although these fiber‐supplemented systems lack the organized mesh architecture of intact scaffolds and therefore exhibit different mechanical properties, the inclusion of fibers still significantly enhances the stiffness and structural integrity compared to fiber‐free hydrogels.^[^ ^213^, ^214^ ^]^

#### 3.2.7 Fiber Reinforcement

In the context of bioprinting, fiber‐enriched bioinks are gaining increasing attention due to their potential to improve printability, shape fidelity, and resolution—factors critical for translating bioprinted constructs into functional applications.^[^ ^215^ ^]^ Even outside the printing context, the addition of fiber fragments can mechanically stabilize hydrogels. Jordan et al.^[^ ^213^ ^]^ for instance, demonstrated that incorporating PCL fibers into a PEO hydrogel increased the composite stiffness by nearly thirteenfold. This effect results from the fibers’ ability to partially absorb and dissipate mechanical forces. The mechanical outcome of such systems depends on several parameters, including fiber concentration, diameter, length, shape, intrinsic material properties, and the nature of fiber–hydrogel interactions.^[^ ^216^ ^]^ Fibers that integrate well with the hydrogel matrix may transfer mechanical loads more effectively, whereas loosely dispersed fibers primarily contribute to passive reinforcement, as shown by Xing et al.^[^ ^217^ ^]^ who crosslinked their aramid nanofibers into the hydrogel to obtain better integration and thus higher hydrogel strength and stiffness. Furthermore, increasing fiber content or using longer fibers may lead to enhanced network density and inter‐fiber contact, but can also promote aggregation, entanglement, and clogging, particularly in extrusion‐based applications.^[^ ^218^ ^]^

#### 3.2.8 Rheological Effects and Printability

In addition to altering the mechanical properties of cast or printed hydrogels, the inclusion of fiber fragments also affects their rheological behavior, which in turn influences the printability of bioinks. Sonnleitner et al.^[^ ^219^ ^]^ demonstrated this by supplementing model inks with cellulose nanofibers and dumbbell‐shaped PCL microfibers^[^ ^220^ ^]^ tracking changes in extrusion performance and post‐printing structural behavior. Two ink types were tested in their study: alginate and Pluronic. The addition of cellulose nanofibers improved shape fidelity in both systems, whereas the PCL microfibers increased the viscosity of the Pluronic ink but also led to a time‐dependent loss of structural integrity after printing. These findings highlight that different fiber types can have distinct and sometimes opposing effects on ink performance. Low‐viscosity bioinks, which are often difficult to print due to poor shape retention and low mechanical stability, can benefit from the incorporation of fiber fragments. The resulting increase in viscosity, improved deposition stability, and enhanced stiffness can significantly improve printability. These effects have been reproduced in various systems. For example, cellulose nanofibers have been shown to improve the printability of alginate‐based^[^ ^214^ ^]^ or pectin‐based^[^ ^221^ ^]^ bioinks. Similar improvements have also been achieved using softer fibers such as silk fibroin nanofibers, which can be blended with a range of hydrogels to enhance shape fidelity.^[^ ^222^ ^]^ A particularly notable example is provided by Choi et al.,^[^ ^223^ ^]^ who added gelatin fibers to an alginate–gelatin bioink. This modification not only improved printability and post‐printing stability but also enabled the fabrication of complex structures such as heart valve and ventricle‐shaped constructs. Post‐seeding, cells successfully adhered to the constructs and aligned along the fiber orientation, demonstrating both mechanical and biological functionality.

#### 3.2.9 Impact on Cell Behavior

The effects of fiber fragments on cellular behavior within hydrogels are largely comparable to those observed in intact fiber membranes embedded in gels. The key difference lies in the more homogeneous distribution of fibers throughout the hydrogel, assuming aggregation is avoided. If the fibers are more attractive to cells than the surrounding hydrogel matrix, cells will preferentially adhere to them. This can accelerate morphological changes, particularly when the hydrogel itself does not provide optimal adhesion cues.^[^ ^224^ ^]^ An illustrative example is provided by Schaefer et al.^[^ ^127^ ^]^ who combined a cell‐repellent recombinant spider silk bioink with collagen‐coated, dumbbell‐shaped fibers. While the presence of fibers initially caused a slight reduction in cell viability, likely due to shear‐induced disruption of early cell–fiber attachments during printing, this short‐term effect was outweighed by a clear long‐term benefit. Over the course of maturation, the fibers provided a more bioactive microenvironment that enabled stable cell adhesion and resulted in higher proliferation compared to fiber‐free controls.

#### 3.2.10 Anisotropy via Fiber Alignment

While casting methods quickly produce structures with randomly oriented, uniformly distributed fiber fragments, bioprinting offers superior control when arranging different cell types and fibers in a spatiotemporally defined manner. For example, varying fiber concentrations within hydrogels can create density gradients that influence ink stiffness and impact cell migration and proliferation. A key advantage of extrusion bioprinting is its ability to align fibers along the printing direction,^[^ ^109^, ^223^, ^225^, ^226^ ^]^ which is especially beneficial for engineering anisotropic tissues like muscle^[^ ^227^ ^]^ or nerve structures^[^ ^115^ ^]^ where directional cues are essential. Post‐printing methods like magnetic orientation can align fibers ^[^ ^228^ ^]^ as for instance done by, Omidinia‐Anarkoli et.al,^[^ ^212^ ^]^ who magnetically aligned their fibers to later facilitate neuronal growth in the direction of the fibers, yet they tend to have lower spatial resolution and are better suited for small‐scale constructs, whilst extrusion‐induced flow alignment during extrusion bioprinting offers a more practical and scalable approach. If such oriented fibers are bioactive, appropriately sized, and embedded in a cell‐supportive ink, cells tend to align along their direction, promoting the formation of organized tissues during and after printing. Prendergast et al.^[^ ^225^ ^]^ demonstrated this by bioprinting aligned hyaluronic acid fibers within a GelMA‐based bioink for meniscal tissue repair. Constructs were printed into a support bath and cultured for 56 days, revealing significantly improved cellular organization in aligned fiber constructs compared to fiber‐free controls. These scaffolds not only enhanced cell and collagen alignment but also exhibited superior tensile moduli, an essential feature for functional meniscal tissue engineering.

#### 3.2.11 Additional Functional Roles

Moreover, fibers can also serve a range of additional functional purposes within hydrogel systems. An innovative application was demonstrated by Lammers et al.^[^ ^229^ ^]^ who incorporated short alginate fibers into fibrin hydrogels as a sacrificial filler material. Following hydrogel crosslinking, the alginate fibers gradually degraded, leaving behind a porous, interconnected network that enhanced nutrient diffusion throughout the construct. Fiber fragments can also be employed as carriers for drug delivery, particularly when the fibers degrade more slowly than the surrounding hydrogel.^[^ ^230^ ^]^ This enables a more sustained release of therapeutic agents over time. Moreover, fiber‐based delivery offers the advantage of localized release. Since cells often preferentially adhere to fibers, bioactive compounds immobilized on the fiber surface can exert stronger effects on cells in close proximity. Given the wide variety of fiber materials and combinations that can be produced,^[^ ^231^ ^]^ there are numerous opportunities to design functionalized fiber–hydrogel composites tailored for specific biomedical applications. These may include controlled drug release, localized stimulation, enhanced diffusion, or spatial patterning of biochemical cues.

## 4 Perspectives

While significant progress has been made in understanding cellular behavior, particularly in 2D cultures, translating this knowledge to 3D environments remains incomplete.^[^ ^232^, ^233^, ^234^ ^]^ However, 3D systems better represent the native cellular environment, with complex cell–matrix and cell–cell interactions that require more advanced models to capture in vivo‐like responses.

Fibrous substrates are central to this, providing both mechanical support and directional cues to guide cellular behavior.^[^ ^145^, ^235^, ^236^ ^]^ In this context, biofabrication offers distinct advantages over traditional tissue engineering using decellularized matrices, which often face issues with donor variability and reproducibility. Fabricated scaffolds ensure consistent quality and standardization,^[^ ^166^, ^237^, ^238^ ^]^ which is vital in systematic studies assessing cellular responses.^[^ ^100^, ^109^, ^115^ ^]^ Moreover, the composition and geometry of these constructs can be precisely adjusted through various deposition and fabrication techniques,^[^ ^226^, ^239^ ^]^ offering an alternative to natural ECM matrices, which lack structural adaptability.^[^ ^95^, ^96^ ^]^ This is also crucial, when designing scaffolds for non‐physiological properties, such as modeling disease‐specific environments or testing cellular responses to controlled environmental changes.^[^ ^240^, ^241^ ^]^

### 4.1 Advantages of Synthetic Fibrous Materials

Biocompatible, biodegradable synthetic polymers that mimic ECM functions are becoming increasingly important for large‐scale production.^[^ ^242^, ^243^, ^244^, ^245^ ^]^ Replacing proteinaceous fibrils like collagen with synthetic alternatives, or using natural materials only as surface coatings,^[^ ^109^, ^122^, ^246^ ^]^ makes fabrication more cost‐effective without compromising cellular functionality. Natural materials, especially animal‐derived ones, present challenges such as batch variability, potential immunogenicity, and inconsistent cell compatibility.^[^ ^247^ ^]^ In contrast, synthetic polymers can be engineered for specific mechanical, chemical, or biological properties and are easier to process.^[^ ^248^ ^]^ Advances in chemical synthesis and biotechnology now enable industrial‐scale production of recombinant proteins or protein‐mimetic polymers in bioreactors. Recombinant materials such as spider silk^[^ ^249^ ^]^ or collagen alternatives^[^ ^250^, ^251^ ^]^ offer high reproducibility and tunability, whereas native materials like animal‐derived collagen are more difficult to process due to compositional variability and sensitivity to processing conditions.

Another key advantage of synthetic fibrous materials is their ease of processing, modification, and deposition compared to natural materials,^[^ ^231^, ^252^ ^]^ enabling simpler optimization and rapid prototyping of scaffold designs. Parameters like fiber diameter, orientation, and surface functionalization can be easily varied and tested without relying on fragile or costly bioactive components. Here for the process of electrospinning allows for diverse fiber morphologies by adjusting process parameters,^[^ ^253^ ^]^ while melt electrowriting enables more complex, precise architectures, including larger fiber diameters and controlled fiber placement.^[^ ^96^, ^100^ ^]^ The combination of these techniques expands design possibilities, creating hybrid scaffolds with hierarchical structures and fine‐tuned mechanical and biological properties.^[^ ^152^, ^210^, ^254^ ^]^

### 4.2 Integration with Microfluidics for Advanced In Vitro Models

The integration of fibrous scaffolds with microfluidic technologies offers a promising frontier.^[^ ^255^, ^256^ ^]^ This hybrid approach combines the structural and biochemical flexibility of fiber‐based ECM analogs,^[^ ^166^, ^257^ ^]^ with the scalability, low cost, and analytical power of microfluidic systems.^[^ ^258^ ^]^ These platforms are ideal for drug screening, personalized medicine, and disease modeling, especially in organ‐on‐chip and body‐on‐chip applications. They also enable 3R‐compatible testing systems, supporting high‐throughput experimentation with minimal sample volumes.^[^ ^259^ ^]^ As 3D tissue models become more complex, automation and precise deposition will be crucial for ensuring reproducibility and accuracy. The convergence of biofabrication, microengineering, and synthetic biomaterials will be key to developing scalable, clinically relevant tissue models for future biomedical applications.

### 4.3 Fibrous Scaffolds for Large‐Scale Tissue Constructs

Fibrous substrates are crucial for large‐scale constructs and organ replacements due to their mechanical stability, ability to enhance cell proliferation, and capacity to guide cell alignment. These properties are essential for replicating the functional architecture of native tissues, especially in skeletal muscle, cardiac muscle, and the nervous system, where anisotropy and cellular organization are key for physiological function.^[^ ^223^, ^260^, ^261^ ^]^ A key question in tissue model development is how much of the final architecture needs to be predefined in the scaffold. In some cases, the scaffold may only need to provide cues for initial organization, allowing cells to self‐organize and remodel during maturation.^[^ ^238^ ^]^ The required architectural precision will vary depending on the tissue type and application.

### 4.4 Standardization and Reproducibility in Scaffold Fabrication

Fibrous substrates must be carefully quantified in terms of geometry (e.g., diameter, distribution, orientation, porosity), chemistry (composition, surface chemistry), physical properties (stability, melting point, swelling), and mechanics (stiffness, elasticity, yield) to ensure reproducibility.^[^ ^262^ ^]^ However, these characteristics can be altered during detachment, storage, and handling, raising concerns about substrate stability, as highlighted by many in the field.^[^ ^95^, ^263^, ^264^ ^]^ To address this, there has been a focus on developing scalable, standardized workflows for both research and industrial applications.^[^ ^166^, ^264^ ^]^ Integrating macroscopic fabrication techniques, like fused deposition modeling (FDM), enhances mechanical stability, improves handling, and ensures more reproducible workflows. This also enables the use of thinner substrates, such as electrospun membranes with thicknesses similar to natural basement membranes.^[^ ^166^ ^]^ These advancements address the long‐standing issue of fibrous scaffold fragility under mechanical stress or dynamic conditions, particularly in bioreactors where handling can compromise structural integrity.

### 4.5 In‐Process Control and AI Integration in Scaffold Production

Another key issue is in‐process control and real‐time analysis during production, whereby the lack of reproducibility and standardized practices is often cited as one of the major barriers to commercializing and improving the accessibility of bioprinting,^[^ ^265^ ^]^ and in part also melt electrowriting.^[^ ^266^ ^]^ This includes challenges like calibrating extrusion rates and adjusting for irregularities or deviations in deposition as they happen.^[^ ^267^ ^]^ To address this, there has been a push to develop optical analytical systems combined with machine learning or AI, which can automatically interpret the results and adjust the printing parameters in real time.^[^ ^215^, ^266^ ^]^ AI tools can be integrated with electrospinning for improved in‐process control. By analyzing datasets on how parameters (e.g., polymer type, solvent, voltage) affect outcomes, the system could predict optimal settings for desired mesh quality, reducing development time.^[^ ^268^ ^]^AI can also generate complex structures, like vessel networks^[^ ^269^ ^]^ or scaffolds that promote specific cellular responses, based on natural analogs.^[^ ^270^ ^]^ This accelerates the modeling process, making complex methods more accessible and interdisciplinary, so they can be used quickly in cell culture labs or by doctors in clinical settings.

Machine learning/AI‐driven predictive systems are particularly useful with scaffolds made from stimuli‐responsive or smart materials. These fibrous materials can sense changes in pH, temperature, electrical stimulation, etc., and respond by altering their shape, color, or releasing a drug, enabling the scaffold to react to internal or external changes.^[^ ^271^ ^]^ To expand on these capabilities computational tools can be used to predict and generate scaffold geometries that adopt the desired conformations before and after stimulation.^[^ ^271^, ^272^, ^273^, ^274^ ^]^ On the other hand, they may also receive and process outputs from cells/tissues interacting with sensory scaffolds^[^ ^274^, ^275^, ^276^ ^]^ and spatiotemporally self‐automate/regulate the scaffold and its maturation. For instance, by monitoring a pH induced local colorimetric change^[^ ^277^, ^278^ ^]^ or by assessing cellular responses to electrical or magnetic stimulation^[^ ^278^, ^279^, ^280^ ^]^ and in turn responding by using other responsive scaffold components, raising the temperature for drug/factor release^[^ ^275^, ^276^, ^281^ ^]^ or adjusting the stimulation rate for instance.

## 5 Conclusion

Fibrous materials play a pivotal role in biofabrication and tissue engineering due to their structural resemblance to the fibrous components of the extracellular matrix, enabling the replication of native tissue architecture and promoting key cellular functions such as adhesion, migration, and differentiation.^[^ ^98^ ^]^ By tuning fiber properties including surface chemistry,^[^ ^131^, ^132^ ^]^ topography,^[^ ^137^, ^140^ ^]^ diameter,^[^ ^121^, ^145^ ^]^ density^[^ ^110^ ^]^ and orientation^[^ ^158^ ^]^ researchers can exert fine control over cell behavior, facilitating the transition from traditional 2D cultures to more physiologically relevant 3D environments.^[^ ^24^ ^]^

**Figure 6:**
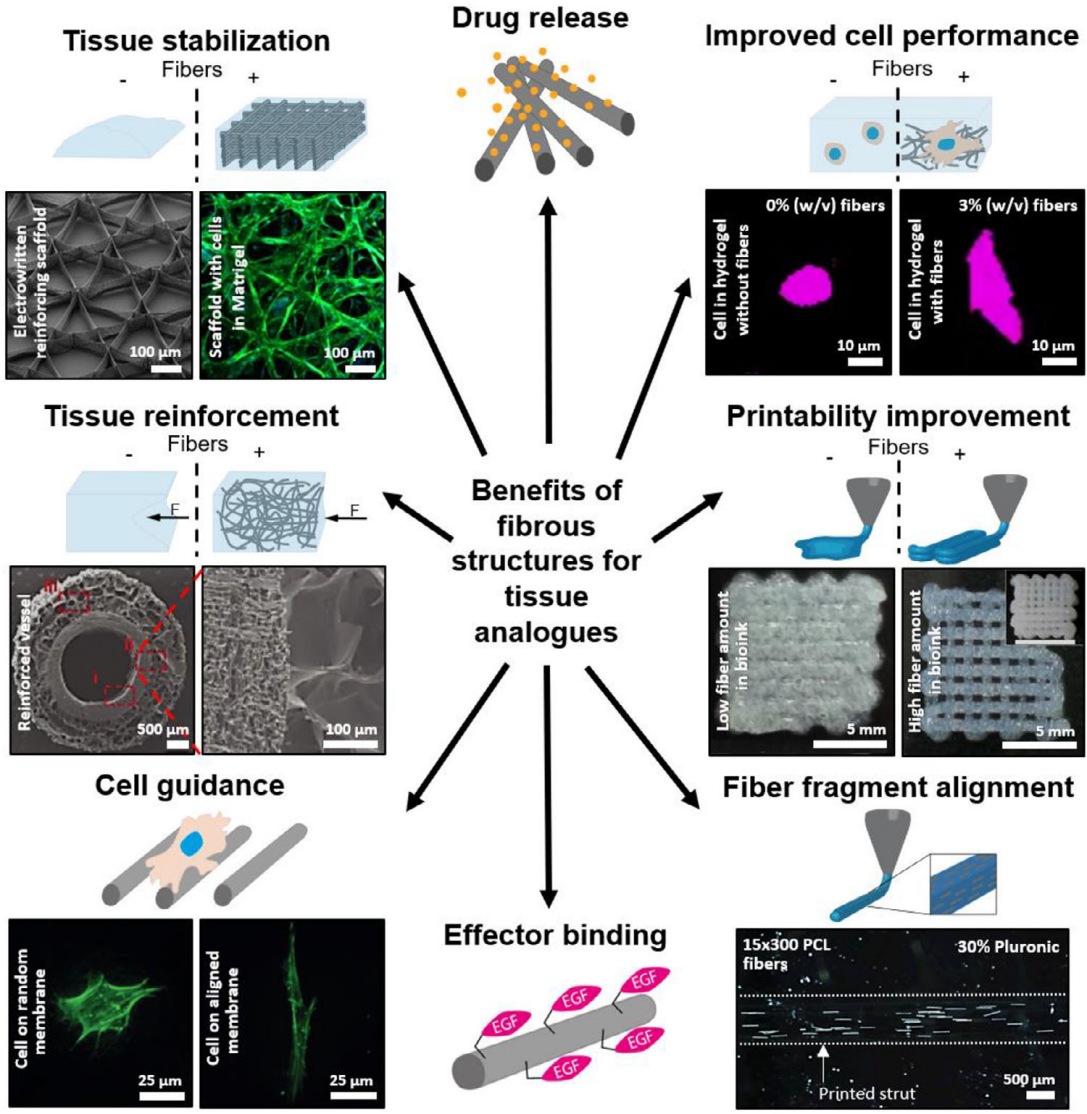
The benefits that fibrous scaffolds or filler materials provide to bioinks in terms of a mechanical and cellular aspect. For stabilization of otherwise too soft hydrogels, meshes can be used to reinforce these, as for example a melt‐electrowritten scaffold used for stabilizing soft hydrogels used to mimic brain tissue, reproduced from Ref. [159] Copyright (2024), with permission from Wiley‐VCH GmbH. Hydrogels can also be further reinforced by fibrous structures, such as exemplarily shown on a vessel‐like structure with an electrospun membrane reinforced inner lumen for resisting greater pressures. Reproduced from Ref. [282] Copyright (2022), with permission from Elsevier. The directionality of cell migration and body polarization can also be influenced by adding anisotropic substrates, well visible when for instance comparing the morphology of a single cell spreading on a random or aligned membrane. Reproduced from Ref. [283] Copyright (2022), with permission from Elsevier. An increased cell performance by adding fibrous components is also possible, as shown by pictures of single cells increasing their surface due to spreading within a recombinant spider silk bioink with 3% (w/v) fibers as opposed to staying rounded in the ink without fibers. Reproduced from Ref. [127] Copyright (2023), with permission from Wiley‐VCH GmbH. The printability of a bioink can also be improved by supplementation with fibrous filler materials, such as in the example showing improved printability with increasing oxidized bacterial cellulose nanofiber amounts in the ink. Reproduced from Ref. [284] Copyright (2020), with permission from Springer Nature. With an adequate fiber type and bioink the flow induced alignment of fibers within a printed strut may also be attained, as shown on an example of 15 × 300 µm^2^ sized PCL fibers in 30% Pluronic. Reproduced from Ref. [109] Copyright (2024), with permission from Wiley‐VCH GmbH. Drugs may also be incorporated into the fibers and released, whilst effectors or drugs may also be bound onto the surface of the fibers to modulate or stimulate cells that contact them.

In 3D matrices, fibrous elements support higher cell densities and improve the mechanical integrity and handling of soft hydrogels, especially when combined with compliant biomaterials.(**Figure** **6**).^[^ ^159^, ^166^ ^]^

Fabrication techniques like electrospinning, melt electrowriting, and fused deposition modeling enable the production of fibrous scaffolds with application‐specific properties,^[^ ^285^ ^]^ advancing the development of structurally^[^ ^286^ ^]^ and functionally mature tissue models.^[^ ^166^ ^]^ Emerging strategies such as fiber‐hydrogel composites,^[^ ^195^ ^]^ and fiber fragment dispersion are particularly promising for inducing precise cell placement^[^ ^237^, ^238^ ^]^ and cellular alignment^[^ ^225^ ^]^ in engineered tissues like cardiac and skeletal muscle.^[^ ^223^, ^227^ ^]^

Despite these advances, critical challenges remain, particularly in 3D systems. Currently, there is still the question of whether the types of fibrous scaffolds that are used are optimal or merely adequate. Addressing this requires systematic and comparative studies that evaluate the impact of fiber characteristics (e.g., diameter, alignment, coating, roughness) in well‐defined 2D models before translating optimized designs to complex 3D environments. Additionally, standardized cellular markers, such as gene expression, protein localization, and morphology, that reliably reflect in vivo tissue function are essential to screen when guiding scaffold optimization. Markers that respond differentially in 3D compared to 2D should be prioritized to refine experimental design.

Ultimately, while fibrous substrates significantly influence cell behavior, they represent only one aspect of functional tissue engineering. A synergistic approach must also consider dynamic biological factors, such as signalling cues, transcriptional regulation, and co‐culture systems, to better mimic the cellular microenvironment. Moving forward, integrating material design with biological complexity will be essential to developing more predictive, reproducible, and translational tissue models.

## Conflict of Interest

The authors declare no conflict of interest.
